# A model of hippocampal spiking responses to items during learning of a context-dependent task

**DOI:** 10.3389/fnsys.2014.00178

**Published:** 2014-09-23

**Authors:** Florian Raudies, Michael E. Hasselmo

**Affiliations:** ^1^Center for Computational Neuroscience and Neural Technology, Boston UniversityBoston, MA, USA; ^2^Center of Excellence for Learning in Education, Science, and Technology, Boston UniversityBoston, MA, USA; ^3^Department of Psychological and Brain Sciences, Center for Systems Neuroscience and Graduate Program for Neuroscience, Boston UniversityBoston, MA, USA

**Keywords:** hippocampus, leaky-integrate and fire neuron, spike timing dependent plasticity, neural network modeling, spiking neural networks

## Abstract

Single unit recordings in the rat hippocampus have demonstrated shifts in the specificity of spiking activity during learning of a contextual item-reward association task. In this task, rats received reward for responding to different items dependent upon the context an item appeared in, but not dependent upon the location an item appears at. Initially, neurons in the rat hippocampus primarily show firing based on place, but as the rat learns the task this firing became more selective for items. We simulated this effect using a simple circuit model with discrete inputs driving spiking activity representing place and item followed sequentially by a discrete representation of the motor actions involving a response to an item (digging for food) or the movement to a different item (movement to a different pot for food). We implemented spiking replay in the network representing neural activity observed during sharp-wave ripple events, and modified synaptic connections based on a simple representation of spike-timing dependent synaptic plasticity. This simple network was able to consistently learn the context-dependent responses, and transitioned from dominant coding of place to a gradual increase in specificity to items consistent with analysis of the experimental data. In addition, the model showed an increase in specificity toward context. The increase of selectivity in the model is accompanied by an increase in binariness of the synaptic weights for cells that are part of the functional network.

## Introduction

Episodic memory involves encoding and remembering the context of an event in which an item or object has been observed. This context could be a specific place, absolute time, or relative time anchored to other events that happened before or after. Our modeling study focusses on the encoding of context in a learned neuronal spiking representation, using interactions of neocortical and hippocampal circuits.

The firing of neurons in the hippocampus is influenced by the context that is relevant to solving a current behavioral task. In a modified T-maze task, two-thirds of the recorded cells in the hippocampus fired differentially as the rat traversed the common stem followed by a left-turn compared to a right-turn (Wood et al., 2000). CA1 neurons during a discrete delayed-nonmatch-to-place task showed selectivity either for the sample phase or the encoding phase of the task (Griffin et al., 2007). Also, hippocampal neurons show prospective and retrospective memory coding, e.g., firing in a differential manner dependent on the start or anticipated end location of a trajectory (Ferbinteanu and Shapiro, 2003). The study presented here focuses on modeling experimental data from a behavioral task that required differential responses to items in different spatial contexts, showing that hippocampal neurons develop selectivity toward specific items in an abstraction of spatial context (Komorowski et al., 2009).

The generation of behavior on the basis of contextual representations appears to depend upon both the hippocampus and medial prefrontal cortex (mPFC). Rats with lesions in the hippocampus show impairments in choosing the correct response to an individual arm in a radial maze based on prior responses. Rats with deactivated mPFC have impaired performance in selecting the correct object (Lee and Solivan, 2008). Rats with lesions in hippocampus have impairments in tasks where object location, object-in-place, or recency information matters, but not for a novel object preference task (Barker and Warburton, 2011). Lesions in either the perirhinal or mPFC led to impairment in tasks involving object-in-place or recency information (Barker and Warburton, 2011). These examples indicate that the coding of context may involve interactions of the hippocampus and mPFC.

How does the encoding of context emerge? To provide a possible answer we model a context-dependent task (Komorowski et al., 2009) and compare the behavior during learning of the simulated rat and model cells with that of the actual experiment. For matched data between simulation and experiment we assume that our model provides a possible mechanism to explain the emergence of context specific coding in cells. This task has four physical locations A1 and A2 in context A and B1 and B2 in context B. At each of these four locations either item X or item Y can appear giving eight stimulus combinations. Each combination is a triplet, e.g., A1X. Reward is given independent of the place but switches the reward-associated item with context. To predict the reward the network has to (i) from a link between item and context to be associated with reward and (ii) has to develop a representation independent of place. This abstraction of place is a non-trivial task because it requires consistent responses despite differences in place, despite the fact that place is usually coded by hippocampal neurons. In general, the sensory system constantly delivers information about place, head direction, geometric information, odors, sequence information, etc. In many experiments, the rat hippocampus appears to form representations that are independent of many of these stimulus variables in order to generate responses that focus on the reward-dependent variables.

Our spiking network model is able to learn the link between item and context while developing independence for place when predicting reward. The item and context selectivity significantly increases while the place selectivity remains constant, as in the data (Komorowski et al., 2009; Figure 5). The correct detection rate increases within 130 trials to about 90%, similar to the data (Komorowski et al., 2009; Figure 6). Our model fits the empirical data while suggesting a new learning method based on replaying rewarded sequences in forward temporal order and non-rewarded sequences in backward temporal order.

## Methods

### Description of the context-dependent task

**Figure 1:**
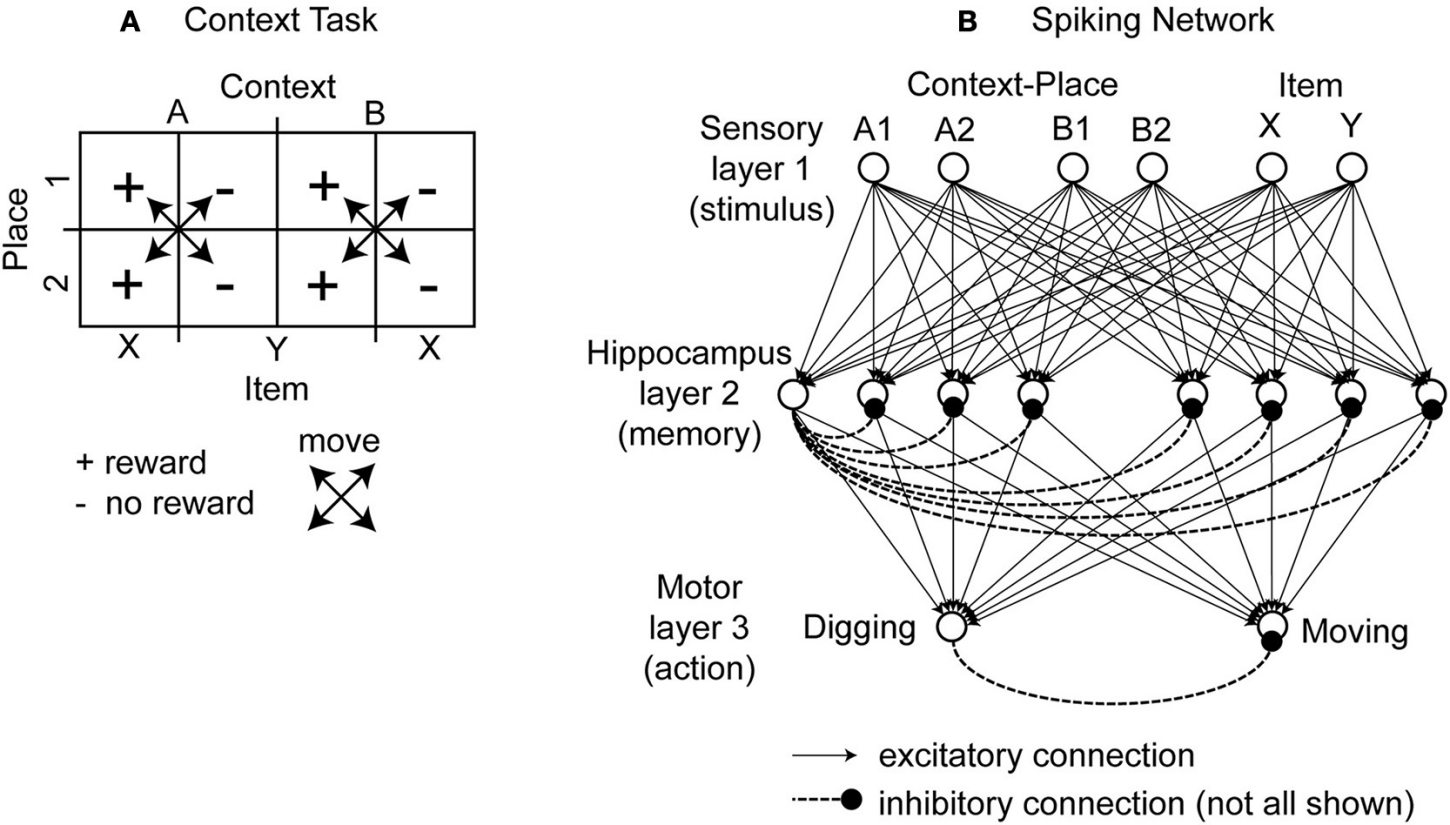
**Illustration of the context-dependent task and schematic drawing of the spiking network. (A)** The context-dependent task has eight triplets defined by context, item, and place. Each triplet is uniquely defined by a context, place, and item, e.g., A1X. Four triplets are rewarded. These triplets are marked by the “+” symbol. And four triplets are without reward. These triplets are marked by the “−” symbol. As the rat moves it always switches place but never context. This is indicated by the crossed arrows. The rat is rewarded for digging (for X and A or Y and B). In unrewarded locations the correct response is to move to a rewarded location. **(B)** The modeled network has a sensory layer with six cells (coding context-place combinations A1, A2, B1, and B2 and items X and Y), a hippocampal layer with eight cells, and a motor layer with two cells (representing digging or moving). Excitatory all-to-all connections exist between layers and inhibitory connections within a layer are to all cells except the cell itself, here drawn only for the 1st hippocampal cell and the 1st output cell.

This task was designed to probe the learning and neural representation of context-item conjunctions (Komorowski et al., 2009). In this task the rat is in one of two boxes, which are different in their visual appearance, which defines the context cue for the rat. We name one box context A and the other box context B. Each of the two boxes has two pots and only one pot has a reward. These pots differ in color, odor, or filling material in which the reward is buried. In abstraction we refer to these two pots as item X and item Y. A pot can appear in one of two spatial places, which we refer to as place 1 and place 2. The use of two different items that could appear in one of two spatial locations in one of two contexts gives eight combinations (Figure 1A). Each of these combinations is called a context-place-item triplet. The triplets A1X, A2X, B1Y, and B2Y are rewarded. No reward is given for the triplets A1Y, A2Y, B1X, and B2X. When switching the context from A to B the reward association changes from item X to item Y.

### Description of the network architecture

To model this task we abstracted from the visual appearance, odors, and tactile input in the form of binary input vectors. In our model sensory signals are delivered through six input cells *n*_*sensory*_ = 6, four to provide context-place information and another two to provide item information. For context-place A1 the 1st input cell is active and for context-place A2 the 2nd input cell is active, and so on (Figure 1B). Similarly, the 5th and 6th input cells are active for item X or item Y, respectively. These six input cells are connected to eight hippocampal cells *n*_*hippo*_ = 8 using the adaptive weights *W*^*exc*^ to define excitatory connections. In our model we used all-to-all connectivity. In addition, the eight hippocampal cells have inhibitory connections *W*^*inh*^ between them, not inhibiting themselves. The eight hippocampal neurons are connected to two motor output cells *n*_*motor*_ = 2 with adaptive weights, again using all-to-all connectivity. These two motor output cells inhibit each other representing lateral inhibition within a structure. We initialized all random weights using uniform noise ranging between 0 and 1. Our model abstracts from these cells and activation pattern required to move a rat or to make a rat dig. Instead one motor cell represents the action “digging” and another motor cell represents the action “moving.” The model rat can only moves between place 1 and place 2—no intermediary places exist. The model rat digs in either place 1 or place 2. The model rat cannot perform any action that would change its context. Instead, context changes randomly between trials. Some trials start the model rat in context A and others in context B. We provide a summary of all model parameters (Table 1).

**Table 1:** **Lists the parameter values that we used in our simulations**.

**Leaky-integrate and fire neuron (LIF)**		
Membrane capacitance	*C*	5.5 × 10^−9^ F
Leaky membrane conductance	*G*_*l*_	10 × 10^−9^ S
Peak membrane potential	*V*_*peak*_	0 mV
Threshold membrane potential	*V*_*th*_	−50 mV
Reset membrane potential	*V*_*reset*_	−70 mV
Standard deviation of Gaussian noise on membrane potential	σ	1 μV per step
**Spike-timing dependent plasticity (STDP)**		
Pre- before post-synaptic spike time constant	τ_+_	10 ms
Pre- after post-synaptic spike time constant	τ_−_	10 ms
Pre- before post-synaptic spike amplitude	*A*_+_	+1.2
Pre- after post-synaptic spike amplitude	*A*_−_	−0.4
Minimum activation for synaptic weight	*w*_min_	0.0
Maximum activation for synaptic weight	*w*_max_	1.0
Time constant for weight adaptation (learning rate)	τ_*w*_	10 ms
**Network parameters**		
Number of sensory neurons	*n*_*sensory*_	6
Number of hippocampus neurons	*n*_*hippo*_	8
Number of motor neurons	*n*_*motor*_	2
Threshold for number of spikes to move	*n*_*th*, *move*_	5
Threshold for number of spikes to dig	*n*_*th*, *dig*_	5
Maximum number of context-place-item-actions for replay	*n*_*replay*_	2
Maximum time interval for a trial	*T*_*trial*_	4000 ms
Maximum time interval for replay	*T*_*replay*_	400 ms
Time increment per simulation step	Δ*t*	0.5 ms
Input current for sensory neuron	*I*_*sensory*_	1.00 nA
Input current for hippocampus neuron	*I*_*hippo*_	0.98 nA
Input current for motor neuron	*I*_*motor*_	0.96 nA

### Description of the network dynamics

All model cells use a leaky-integrate and fire model. We use this model to express the spiking dynamics. The membrane is modeled through a capacitance C that has the potential *V*_*i*_, which is driven by the input current *I*_*j*_ while leaking current through a leaky channel of conductance *G*_*l*_. The membrane has the resting potential *V*_*reset*_. Small fluctuations of the membrane potential are simulated by adding a noise term η. Note that η denotes the random variable with η ∈ *N*(μ, σ) drawn from a Gaussian distribution *N* with mean value μ, here μ = 0, and standard deviation σ. Combining these properties in a dynamic equation gives:

(1) $$C\frac{dV_{i}}{dt}=G_{l}(V_{i}-V_{reset})+I_{k}+\eta$$

with *i* = 1… *n*_*k*_ and *k* ∈ {*sensory*, *hippo*, *motor*}. So “*i*” indexes model cells of a specific layer, either being the sensory layer, the hippocampal layer, or the motor layer, which is indicated by index “*k*.” In addition to this simple model dynamics of Equation (1) we set the membrane potential *V*_*i*_ for one time step to the value *V*_*spike*_ whenever *V* is above the threshold voltage *V*_*th*_. After that one time step the membrane potential *V*_*i*_ is set to the reset voltage *V*_*reset*_. In our simulation we use the Euler method to solve the ordinary differential equation (ODE), Equation (1), thus, one time step equals one iteration and simulates Δ*t* = 0.5 ms. The input current for the sensory layer is *I*_*sensory*_ = 1.00 nA, that for the hippocampal layer *I*_*hippo*_ = 0.98 nA, and that of the motor layer *I*_*motor*_ = 0.96 nA. This gradual decrease in input current leads to an ordered succession of spikes with small time intervals in between.

For the weight adaptation we use the spike-timing dependent plasticity (STDP) rule for synaptic modification (Bi and Poo, 1998). Weights between the sensory layer, the hippocampal layer, and the motor layer are modified. This rule uses the relative timing Δ between the pre-synaptic and post-synaptic spike. If the pre-synaptic spike arrives before the post-synaptic spike, this gives a positive time difference Δ > 0, which leads to a synaptic long term potentiation (LTP). If the pre-synaptic spike arrives after the post-synaptic spike, this gives a negative time difference Δ < 0, which leads to synaptic long term depression (LTD). Such effects happen within a small time window of ≈20 ms. The amplitude for depression *A*_−_ is ≈1/3 of the amplitude for potentiation *A*_+_. We limited the dynamic range of the weights between *w*_min_ = 0 and *w*_max_ = 1. The weight change is defined by the ODE:

(2) $$\begin{array}{l} \tau_{w}\frac{dW_{ij}^{exc}}{dt}=\left(w_{\max}-W_{ij}^{exc}\right)\times A_{+}\exp\left(-\Delta /\tau_{+}\right)\\ \;-\left(w_{\min}-W_{ij}^{exc}\right)\times A_{-}\exp\left(+\Delta /\tau_{-}\right) \end{array}$$

with *i* = 1… *n*_*k*_, *j* = 1… *n*_*l*_, *k* ∈ {*sensory*, *hippo*, *motor*}, *l* ∈ {*sensory*, *hippo*, *motor*} and *k* ≠ l. Indices “*i*” and “*j*” index cells from the two connected layers, e.g., the sensory layer with the hippocampal layer. The time constants τ_*w*_, τ_+_, and τ_−_ control the weight adaptation, exponentially decaying influence of LTP and LTD, respectively.

Activity of cells flows between layers through the excitatory weights *W*^*exc*^ and inhibitory weights *W*^*inh*^. At the receiving terminal we use a winner take all rule to generate a current pulse of *I*_*hippo*_ = 0.98 nA for cells in the hippocampal layer or *I*_*motor*_ = 0.96 nA for cells in the motor layer. This is expressed by:

(3) $$\begin{array}{l} I_{j^{*}}=I_{k}\text{ if }j^{*}=\arg\;\max_{j}\{\sum_{i\;=\;1}^{n_{k}}\left(V_{i}-V_{reset}\right)\;W_{ij}^{exc}\\ \;-\sum_{\begin{array}{c} i\;=\;1\\ i\;\neq \;j \end{array}}^{n_{k}}\left(V_{i}-V_{reset}\right)\;W_{ij}^{inh}\}\text{ and }j=1\ldots n_{l}. \end{array}$$

Otherwise the input current *I*_*j*_ is set to the value of zero. Again, *k* ∈ {*sensory*, *hippo*, *motor*} and *l* ∈ {*sensory*, *hippo*, *motor*}.

### Simulating the network

We simulated the described network for 100 runs, where each run consists of 130 trials. A trial itself consists of two phases. During the first phase the model rat explores the environment. In our simulation the model rat moves between the pots or digs in one of these pots. This first phase ends with digging and can consist of multiple moves (which happens particularly during initial learning), and one digging event that terminates the trial. Taking these actions together in their temporal order defines an action sequence.

After the first phase of a trial, which generated one action sequence, the second phase of the trial replays this action sequence. Rewarded action sequences are replayed in forward temporal order. Combined with the STDP learning rule this replay enhances such action sequences. They are more likely to be chosen in the future. Non-rewarded action sequences (that ended with digging in a non-rewarded pot) are replayed in backward temporal order. Combined with the STDP learning rule such reply discourages the future use of such action sequences.

**Figure 2:**
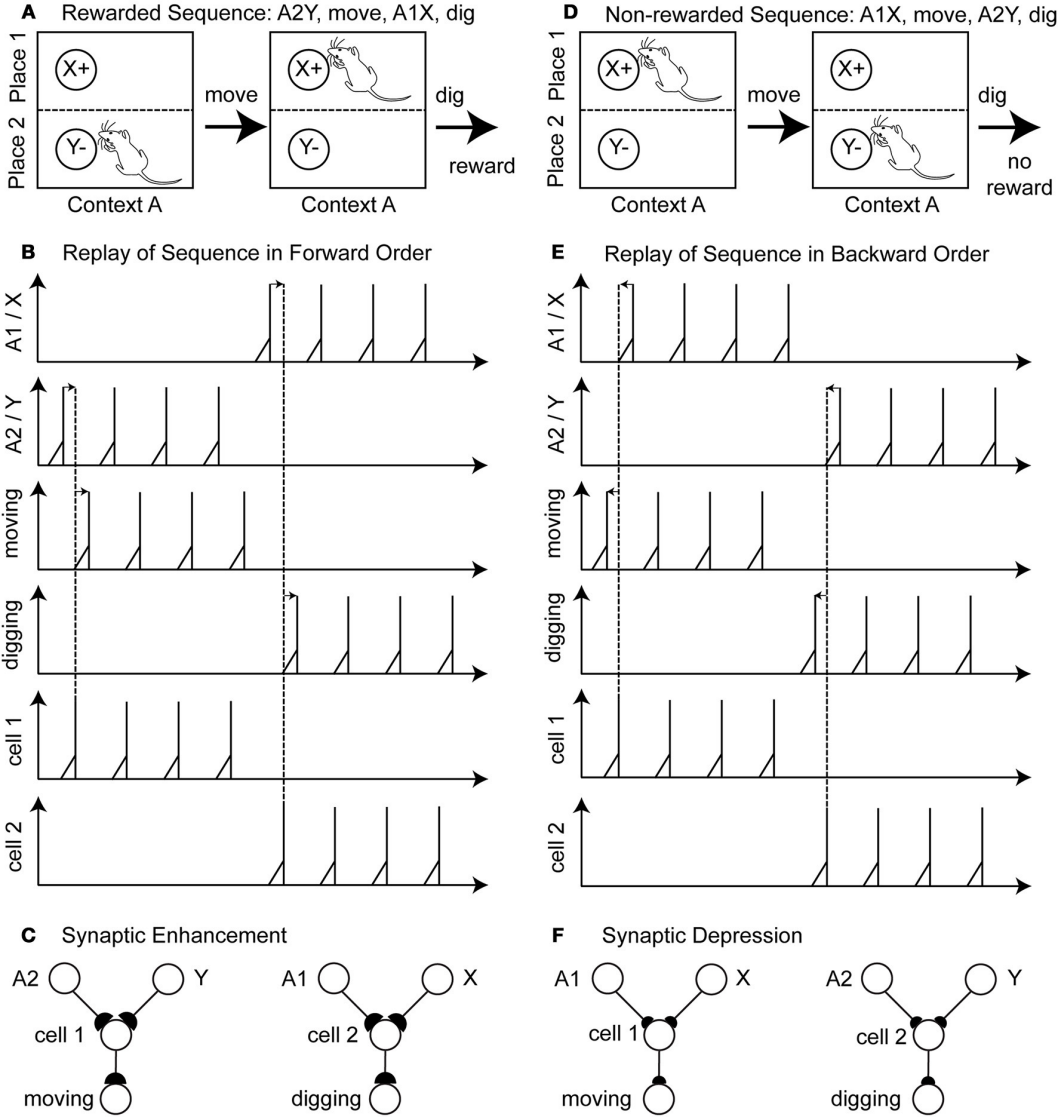
**Illustration of the proposed learning scheme: Rewarded action sequences are replayed in forward order and non-rewarded action sequences are replayed in backward order**. **(A)** Shows the rewarded action sequence: A2Y, move, A1X, dig, and receiving reward. **(B)** When replaying this sequence in forward temporal order spiking appears 1st in the sensory cells of place A2 and item Y, 2nd in the hippocampal cell 1 or 2, and 3rd in the output cell encoding the action “moving” or “digging.” **(C)** This spiking in forward temporal order with pre-synaptic spikes occurring before post-synaptic spikes leads to synaptic enhancement—denoted by larger synaptic buttons. **(D)** Shows the non-rewarded action sequence: A1X, move, A2Y, dig, and receiving no reward. **(E)** When replaying this sequence in backward temporal order spiking appears 1st in the output cells, 2nd in the hippocampal cell 1 or 2, and 3rd in the sensory cells. **(F)** Post-synaptic spikes appear before pre-synaptic spike, which leads to synaptic depression—denoted by the smaller synaptic buttons.

An example of a rewarded action sequence is A2Y, move, A2X, dig, and receive a reward (Figure 2A). Initially the sensory cells fire to encode place A2 and item Y. In the example in the figure we assume that learning during phase 1 has already occurred so that there is already the correct connectivity established to active the hippocampal cell 1 that causes synaptic activation of the cell encoding the action “move.” This cell fires several times, resulting in the action “move” being executed by the rat. Then after moving, the rat now senses the place A1 and item X and due to the established connectivity this activates a hippocampal cell 2 that activates the cell coding the action “dig.” The rat receives a reward. During replay, the last two components of the rewarded action sequence (A2Y, move) and (A1X, dig) are separately reactivated in the network by input current of different magnitudes to different cells that causes spiking in a specific order. This reactivation causes first spiking in the sensory neurons representing A2Y, second in the hippocampal cell 1, and third in the action cell coding “move” (Figure 2B—forward order is indicated by left-to-right directed arrows). After several repetitions, there is also replay of the second segment involving sensory cells for A1X, hippocampal cell 2 and the action cell coding “dig.” Because of the forward order of replay the synaptic weights between the sensory cells A1, A2, X, Y, the hippocampal cell 1 and 2 and the action cells encoding move or dig are potentiated (Figure 2C—potentiation is depicted by larger buttons).

A non-reward action sequence is A1X, move, A2Y, dig (Figure 2D). When replayed after absence of reward, the model performs replay in reverse temporal order of the behavior with spiking, first in the action cell coding “dig,” second in the hippocampal cell 1, and third in the stimulus cells A2 and Y (Figure 2E—backward order is indicated by right-to-left directed arrows). Then the other part of the non-rewarded sequence is replayed in reverse order as action cell “move” followed by hippocampal cell 2 followed by sensory cells A1 and X. This backward order leads to a depression of the synaptic weights between the sensory cells A1, A2, X, Y, the hippocampal cells 1 and 2 and the action cell encoding move or dig (Figure 2F—depression is depicted by smaller buttons). In this example we abstracted from the time these action sequences take.

Typical action sequences are ≈1200 ms long and can take *T*_*trial*_ = 4000 ms at the most while replay takes *T*_*replay*_ = 400 ms at the most per action sequence. We replay up to two state-actions of a sequence, *n*_*replay*_ = 2. Replay of an action sequence happens much faster than the actual time to undergo such an action sequence. This replay allows learning much faster than performing the actual action sequence. During replay the fluctuations of the membrane potential are kept at zero (σ = 0). For reasons of efficiency we only evaluate spikes within a window of 10 ms. Spikes that are further apart have only small effects.

To simulate attenuation of repetitive behavior we added an adaptive threshold mechanism for actions. Initially, thresholds for the move action *n*_*th*, *move*_ and that for the digging action *n*_*th*, *digging*_ are both set to a value of five, meaning it takes five spikes for that output cell before the action is executed. However, to discourage a behavior of only moving between the pots we decrease the digging threshold by one with each move. Thus, after at most five movements digging occurs. After executing digging the threshold for digging is reset to the original number of five. Similarly, we reduce the threshold for moving with each digging by one and after moving the threshold for moving is reset to five.

We adjusted the leaky conductance *G*_*l*_ and the value of the input current *I*_*j*_ to define an inter-spike interval of 124 ms and, thus, spiking occurs at a frequency of 8.1 Hz. This spike frequency is within the theta band, which we assume is used during replay and adjustment of the synaptic strength through STDP.

### Definition of place selectivity, item, and context selectivity

To characterize the selectivity of model cells we use the definition of the selectivity index (SI) (Moody et al., 1998; Komorowski et al., 2009). By *n* we refer to the number of stimulus events, λ_*i*_ denotes the firing rate in response to the *i*th stimulus event for a single cell, and λ_*pref*_ denotes the preferred stimulus event of that same cell. We compute λ_*pref*_ taking the maximum firing over all stimulus events for each cell separately. Then the

(4) $$SI=(n-\sum_{i\;=\;1}^{n}\lambda_{i}/\lambda_{pref})/(n-1).$$

For the place selectivity *n* = 4 because the context-dependent task has four physically different places. These are A1, A2, B1, and B2. When we calculated place selectivity we combined the firing for the rat encountering item X and Y when the rat was in each of these four places by computing the mean of firing for item X and Y. For item selectivity *n* = 2, because there are only two items in the context-dependent task. We calculate the mean firing for the set A1X, A2X, B1X, and B2X as well as the set A1Y, A2Y, B1Y, and B2Y. The 1st set includes all triplets with item X and the 2nd set includes all triplets with item Y. For context selectivity, again *n* = 2, because there are only two contexts. In this case we calculate the mean firing for A1X, A2X, A1Y, and A2Y as well as B1X, B2X, B1Y, and B2Y. The 1st mean includes all triplets with context A and the 2nd mean includes all triplets with context B.

For all SIs we calculated the mean and standard error of firing over four 30 trial blocks starting from trial one. The 1st block covers trials 1–30, the 2nd block covers trials 31–60, the 3rd block covers trials 61–90, and the 4th block covers trials 91–120. For the calculation of these selectivity indices we used only hippocampal cells that were part of the functional network. Cells were identified as part of the functional network if their synaptic strength to one of the output cells was greater than 1.0–10^−6^. In most cases we identified four hippocampal cells as being part of the functional network.

### Definition of binariness for synaptic weights

To quantify the functional connectivity we analyzed the binariness of the synaptic weights between the sensory layer and the hippocampal layer. For network configuration these are 6 × 8 weights. As before, we only included hippocampal cells, which are part of the functional network, into the analysis. Assume weights are identified by *W*_*ij*_, then their binariness is

(5) $$B_{ij}=4{\left(W_{ij}-0.5\right)}^{2}$$

with *i* = 1… *n*_*sensory*_, *j* = 1… *n*_*hippo*_. Equation (5) maps the extreme points 0 and 1 to 1 and the mid-point 0.5 to 0. Intuitively, the binariness is large if weights are either close to 0 or 1 and binariness is small if the weights have values close to 0.5.

## Results

In brief, Komorowski et al. (2009) found that place-item selectivity increased with learning and was positively correlated with the correct behavioral response while place selectivity remained constant. The context-dependent task was learned in about 100 trials and rats reached about 80% to 90% correct behavioral response. After training was complete about half (52%) of the initially place selective cells had converted into conjunctive place-item cells while the other part remained place selective. We proposed a model network that is able to learn the context-dependent task in about 100 trials with a ≈90% correct detection rate. During learning a compact representation of the rule associations is formed for the hippocampal cells grouping sensory triplets and output actions whenever possible. This grouping led to about half the cells forming place-item selectivity while the other half remained place-selective. We provide Matlab 7.12.0.635 (R2011a) code to reproduce all the result figures at the journal's website (Supplementary Material).

### Synaptic weights are adapted to learn the context-dependent task

**Figure 3:**
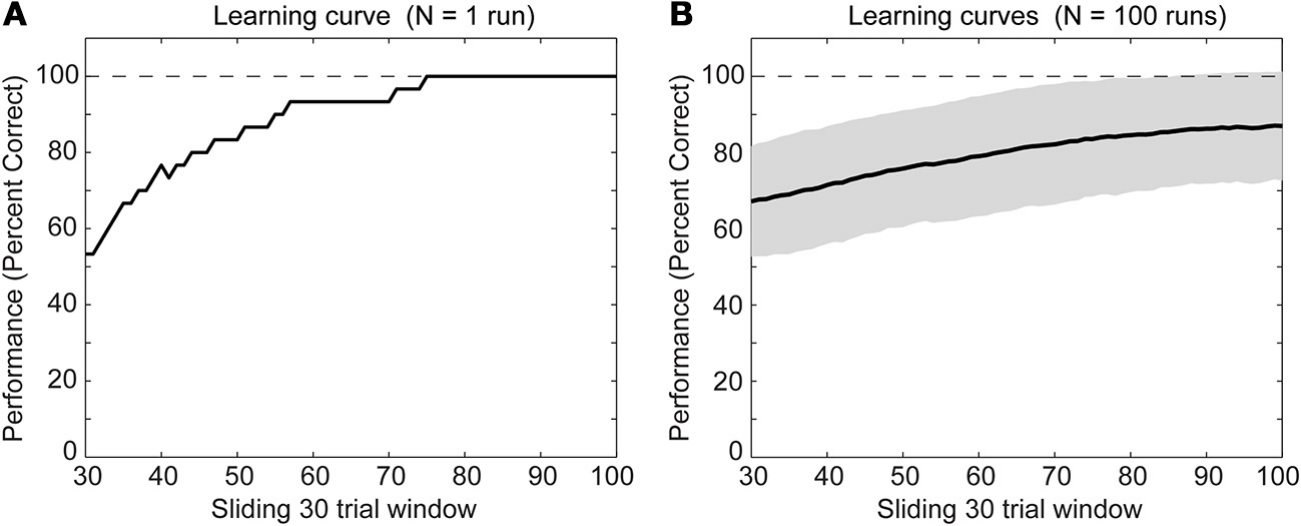
**Behavioral performance during successful learning of the context-dependent task (A) for a single run and (B) for *N* = 100 runs through learning of the task**. The solid line shows the mean and the gray area behind the solid line shows ± one standard deviation from the mean. For both panels we calculated mean values over a sliding window of 30 trials. Notice that we neither show the first nor last 30 trials because of boundary effects caused by using the sliding window.

We simulated a single run of our network with randomly initialized weights. After several trials the rat model learned the task of digging in context A when sensing the item X or digging in context B when sensing the item Y (Figure 3A). Other random initializations of weights—we tested *N* = 100 runs—led to the learning of the task as well (Figure 3B). For some initializations the task was learned at a slower rate and had not reached 100% correct detections after 100 trials. The speed of learning is controlled by the parameters *A*_+_, *A*_−_, and τ_*w*_. We chose these parameters to match the behavioral data of the context-dependent task (Komorowski et al., 2009).

In our network we were able to identify the functional contributions of model cells. Naïve connectivity uses one hippocampal cell per possible context-place-item triplet. For these three input variables each with two possible values, we get eight combinations which is the same number that we provided as hippocampal cells in the model. For instance, we could chose cell 1 to represent A1X, cell 2 to represent B1Y, and so on (see Table 2—naïve connectivity). Since A1X represents a rewarding context-place-item triplet, cell 1 is wired to the output action “digging.” Since the A1Y triplet receives no reward, cell 5 is wired to the output action “moving.” This naïve connectivity scheme leads to the correct function, always digging for reward in the correct place and context. However, such explicit representation suffers from a combinatorial explosion. Assume there are *n*_*var*_ many input variables and each can take on *n*_*value*_ values. Such naïve connectivity requires (*n*_*state*_)^*n*_*var*_^ cells, which grows very fast.

**Table 2:** **In the first part shows the naïve connectivity using the simplest wiring scheme, which has no grouping of similar context-place-item triplets**.

	**Cell 1**	**Cell 2**	**Cell 3**	**Cell 4**	**Cell 5**	**Cell 6**	**Cell 7**	**Cell 8**
**NAÏVE CONNECTIVITY HAS NO GROUPING**								
A1	1	0	0	0	1	0	0	0
B1	0	1	0	0	0	1	0	0
A2	0	0	1	0	0	0	1	0
B2	0	0	0	1	0	0	0	1
X	1	0	1	0	0	1	0	1
Y	0	1	0	1	1	0	1	0
Dig	1	1	1	1	0	0	0	0
Move	0	0	0	0	1	1	1	1
Function	A1X	B1Y	A2X	B2Y	A1Y	B1X	A2Y	B2X
**LEARNED CONNECTIVITY SHOWS GROUPING**								
A1	0.22	1.00	0.44	0.63	1.00	0.02	0.00	0.48
B1	0.43	0.26	0.16	0.58	0.04	1.00	0.52	1.00
A2	0.21	1.00	0.62	0.60	1.00	0.50	0.32	0.00
B2	0.65	0.19	0.27	0.27	0.17	1.00	0.50	1.00
X	0.24	0.08	0.29	0.28	1.00	0.02	0.18	1.00
Y	0.43	1.00	0.30	0.25	0.13	1.00	0.50	0.42
Dig	0.34	0.35	0.77	0.48	1.00	1.00	0.41	0.60
Move	0.62	1.00	0.52	0.57	0.41	0.41	0.60	1.00
Function	None	A1/2,Y	None	None	A1/2,X	B1,2/Y	None	B1,2/X

*The second part shows the learned connectivity, which shows grouping of context-place-item triplets into pairs including both places 1 and 2*.

We noticed that the learned connectivity uses a different encoding scheme. Such a scheme combines or merges context-place-item triplets that all map to the same required action. In our case for both A1X and A2X triplets reward is received and, thus, both triplets map to the output action “digging.” Thus, these two triplets can be grouped together and represented through a single cell. Similarly, A1Y is merged with A2Y for the action “moving.” B1Y is merged with B2Y for the action “digging.” And, B1X is merged with B2X for the action “moving” (see Table 2—learned connectivity). A grouping of the four triplets A1Y, A2Y, B1X, and B2X for the action “digging” is not possible because such paring would also include A1X, A2X, B1Y, and B2Y, which map to the action “moving.” Our learning method chose the maximum grouping of triplets. This grouping of triplets or in general states is similar to the minimization of Boolean functions, which can be solved by the Quine-McCluskey algorithm (McCluskey, 1956).

Using this minimized representation only four out of eight hippocampal cells are required to implement the correct function. We describe these four cells as part of the functional network because they contribute to the output function, which the network realizes. The other four hippocampal cells are not part of the functional network.

### Item and context selectivity emerges while maintaining place selectivity

**Figure 4:**
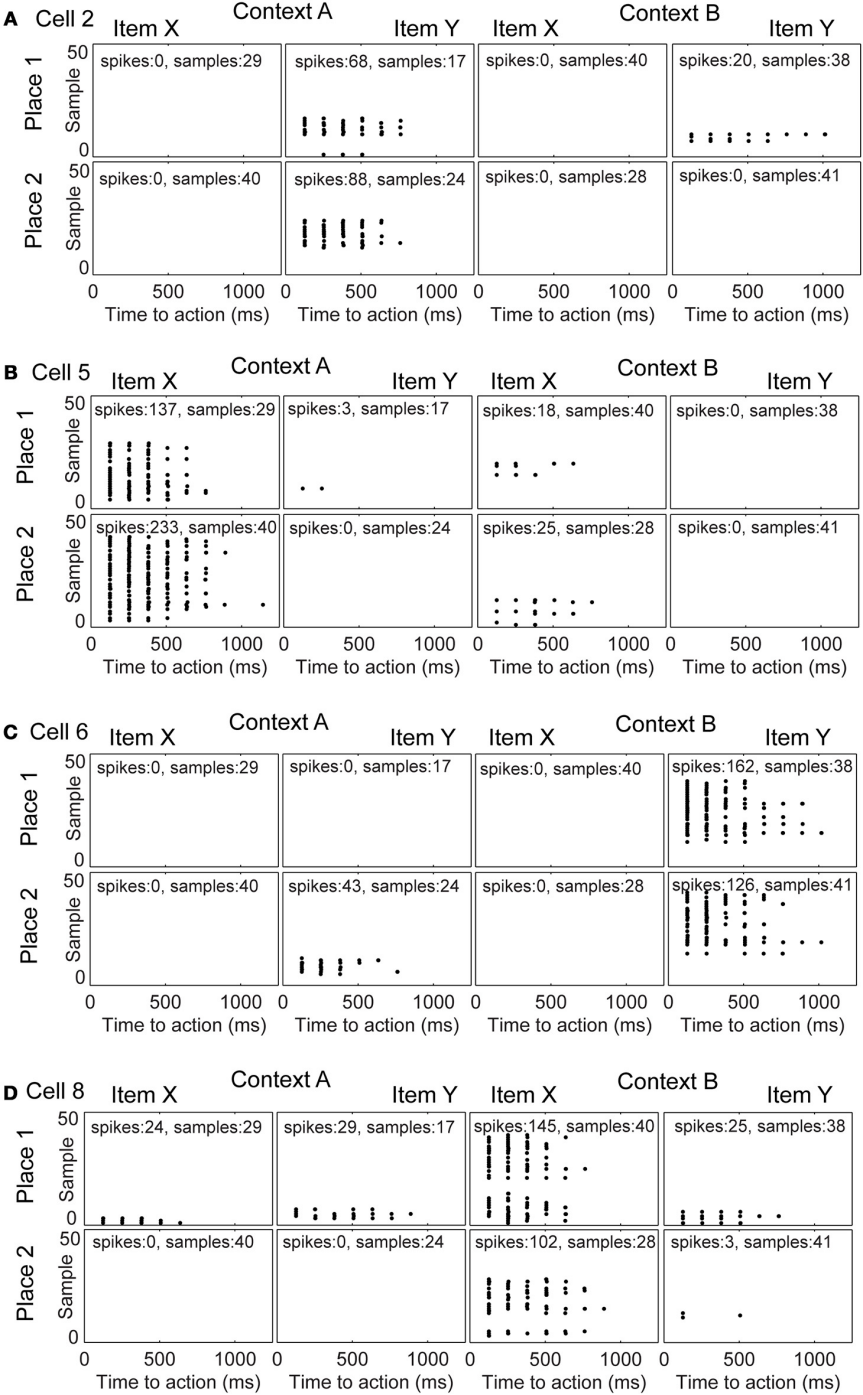
**Evolution of spike patterns for four hippocampal cells, which are all part of the functional network used to successfully perform the context-dependent task**. In the raster plots spikes are depicted by black dots occurring in time relative to the onset of an action. High place selectivity occurs if a cell fires in only the row “Place 1” or “Place 2.” High item selectivity occurs if a cell fires for only one item and not the other regardless of context or place. High context selectivity appears if a cell fires only within one context and not the other context. Firing patterns are shown for **(A)** cell 2, **(B)** cell 5, **(C)** cell 6, and **(D)** cell 8. The sample index—these are the rows in the spike raster plots—appear in the same temporal order as they have been recorded per panel. Because plots are shown for individual samples in the random task, it is not possible to compare across panels in terms of their relative order or timing.

We studied the evolution of spike patterns during the learning of the context-dependent task. Here, we focused only on modeled hippocampal cells, which were part of the functional network. As noted, typically only four out of eight hippocampal model cells are part of the functional network. In one specific run, the same as we showed the learning curve for Figure 3A, the hippocampal cells that are part of the functional network have indices 2, 5, 6, and 8 (Figure 4). Toward the end of the simulation cell 2 fires selectively for item Y in context A, regardless of the place the item Y appears in Figure 4A. Cell 5 fires selectively for item X in context A (Figure 4B). Cell 6 fires selectively for item Y in context B (Figure 4C). Cell 8 fires selectively for item X in context B (Figure 4D).

**Figure 5:**
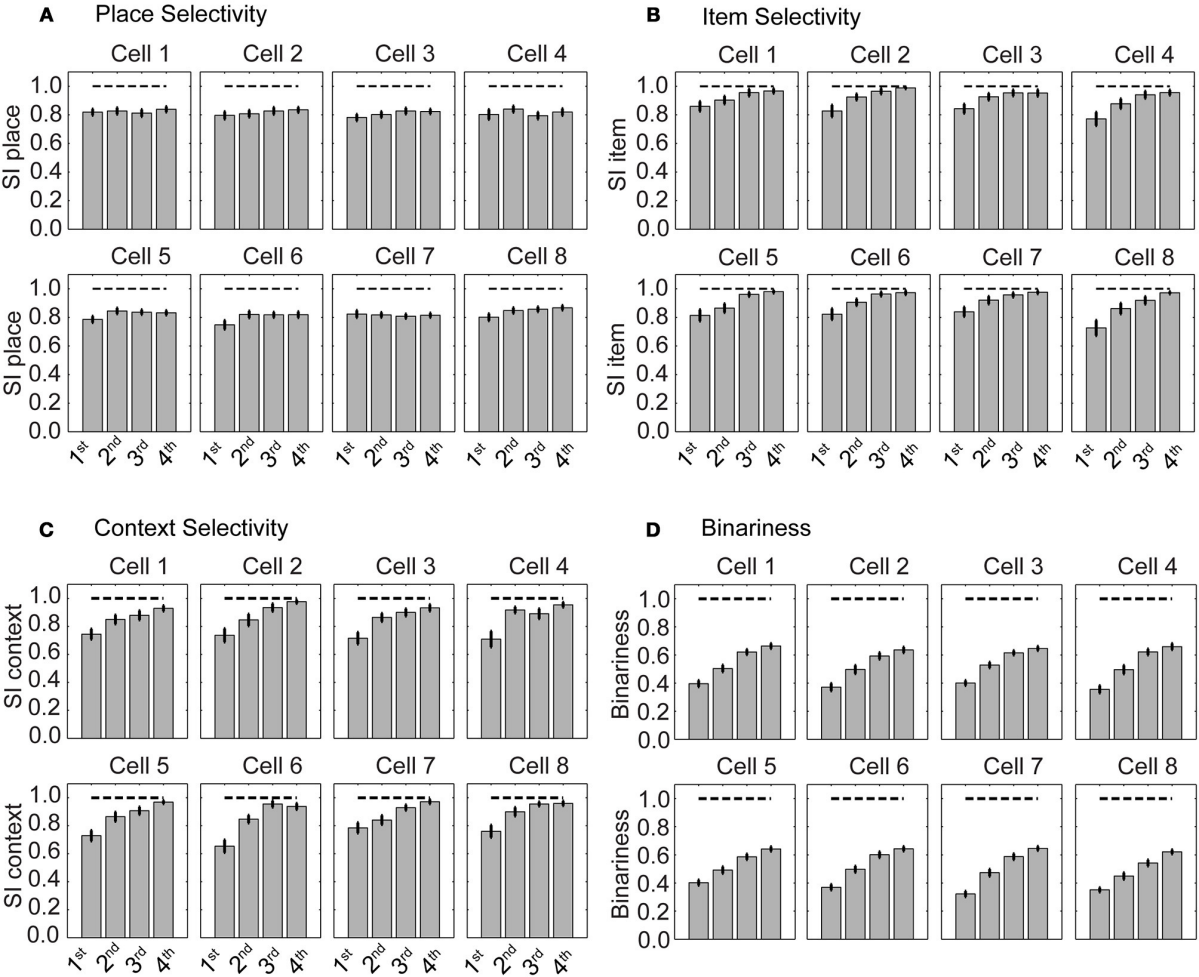
**Selectivity for place is maintained and selectivity for item increases during learning**. **(A)** Selectivity index (SI) for place, **(B)** for item, and **(C)** for context. **(D)** Binariness of synaptic weights between the sensory layer and hippocampal layer. The gray bars show the mean SI calculated from all cells that are part of the functional network. Superimposed vertical black lines denote ± one standard error of the mean.

We evaluated cell selectivity more rigorously using *N* = 100 runs—with different weight initialization and random noise. The mean SI for place is ≈0.8 and stays constant evaluating four successive blocks of 30 trials each (Figure 5A). The 1st block contains the first 30 trials, the 2nd the next 30 trials, and so on. The mean SI for item selectivity starts at ≈0.8 and increases to ≈1.0 over the same successive four blocks of 30 trials each (Figure 5B). The mean for context selectivity starts at ≈0.7 and increases to ≈1.0 over four blocks (Figure 5C). Item and context selectivity between the first and last 30 trial block is significantly different (*P* = 0.01) and that for place selectivity is not different for all cells except a single hippocampal cell (cell 8) (*P* = 0.05). Thus, place selectivity is maintained while item and context selectivity increases during learning.

Weights become more binary as the selectivity increases (Figure 5D). Binariness starts at a value of ≈0.4 for the 1st block of 30 trials and has a value of ≈0.7 for the last block of 30 trials. Comparing the 1st with the last block the binarininess has significantly changed (*P* = 0.01). Thus, function and connectivity emerge jointly in our model network.

The alteration in selectivity can be explained by the definition of the SI and the learned connectivity (see Table 2—learned connectivity). Place selectivity compares the firing for one place with that of all other three places combining responses for different items. At the end of the training a hippocampal cell fires for one item-context in two out of four places but not at the same rate, which leads to the SI of ≈0.8. Item selectivity compares the firing from the set of four triplets A1X, A2X, B1X, and B2X, which all include item X, with the set of four triplets A1Y, A2Y, B1Y, and B2Y, which all include item Y. In our case at most two out of these four triplets have high firing rates, which combined by calculating the mean yield an even more robust response. This high firing rate is contrasted with the low firing rates or absent firing in the other context. Following the definition of the item selectivity this gives an SI of ≈1.0. Similarly for the context the firing from the set of four triplets A1X, A2X, A1Y, and A2Y is contrasted with the firing from the set of triplets B1Y, B2Y, B1X, and B2X. Again one group of triplets contains high firing, which is contrasted with the low or absent firing in the other group. This leads to an SI of ≈1.0. Taking these SIs together, we notice that cells are selective toward item and context but not place.

### Identifying neurons contributing to the behavioral function

In our spiking network model we were able through the connectivity (Table 2) to infer which neuron was part of the behavioral function. Four out of eight hippocampal neurons were part of the behavioral function. Cells that were not part of the function had typically low firing rates, or remained silent during the duration of the simulation consistent with data showing only about 30% of hippocampal cells are active in a given environment (O'Keefe and Nadel, 1978; Wilson and McNaughton, 1993). Cells that were part of the function spiked frequently at ≈8 Hz (Figure 2). Initially, these cells tended to code for place or item, but then shifted toward a coding for item in combination with place (e.g., Figure 4B fires for item X in context A place 2, whereas cell 8 fires for item X in context B place 1). Which hippocampal cells would be part of the functional network depended upon the random weight initialization and the noise in the membrane potentials. As we increased the number of hippocampal cells to 12 and increased the duration of learning from 130 trials to 200 trials, 4–6 cells were part of the behavioral function. Triplet and duplet coding appears in this case. We increased the number of trials because there are more possible connections to be learned for 12 instead of eight hippocampal cells.

### Learning of the model

We proposed a learning mechanism that uses forward and backward replay based on physiological data showing both directions of replay (Foster and Wilson, 2006; Diba and Buzsáki, 2007; Davidson et al., 2009). Action sequences that take seconds in real time show spiking within a few milliseconds during replay. This spiking during replay changes synaptic connectivity through STDP. Notice that STDP is not effective during the time span of an action sequence during behavior. Long term depression (LTD) was necessary to make the model learn the context-dependent task. In addition, the relative magnitude between LTD and LTP also matters for successful learning. Leaving all other parameters constant (see Table 1) we changed *A*_+_ and *A*_−_ to 1.0 and −1.0 in simulations and the task performance of the simulated rat remained at ≈50%.

## Discussion

We presented a spiking neural network that learned context-specific rules using an efficient internal representation by grouping overlapping input states with the same output actions. Model performance in the task matches the behavioral performance of the rat (Komorowski et al., 2009). The model and the rat learned the task within ≈100 trials, with some variability (Figure 3). In the model the selectivity for items increases while that for place remains constant (Figure 5) consistent with previous experimental data.

### Relationship to anatomical structures mediating context learning

The model addresses the neural activity observed in hippocampal region CA1 during learning of behavioral responses to specific items that depend upon the overall context but not the spatial location of the item (Komorowski et al., 2009). The structure of the model is relatively abstract, but was designed to represent the interaction of hippocampal representations in region CA1 with sensory input and motor output (Griffin et al., 2007). The hippocampus receives convergent input about both sensory input and motor output from a hierarchy of brain regions that converge upon the entorhinal cortex (Canto et al., 2008). Layer III of the entorhinal cortex provides direct input to region CA1 of the hippocampus, and region CA1 projects back to deep layers of entorhinal cortex directly or via the subiculum. The inputs to entorhinal cortex include the mPFC, which contains neurons that respond on the basis of both sensory input and motor output. Neurons in the hippocampus have also been shown to respond to sensory stimuli (Wood et al., 1999) and motor outputs (Eichenbaum et al., 1989; Wiener et al., 1989). Thus, the necessary physiological information for the learning presented here is present in the hippocampal formation. In terms of the mechanism involving replay, this could be conceived as involving neurons in the hippocampus that have greater interactions with specific cortical regions. Thus, the sensory input could correspond to neurons in region CA1 that get a strong sensory input via the entorhinal cortex (e.g., Eichenbaum et al., 2007; Deshmukh et al., 2010). The motor output could correspond to neurons in region CA1 that have strong bidirectional connectivity with entorhinal neurons receiving representations of spatial actions from mPFC (Hyman et al., 2010) or parietal cortex (Nitz, 2012).

### Comparison to other proposed learning mechanisms

Hippocampal models by Marr (Marr, 1971; O'Keefe and Nadel, 1978) and others focus on the encoding and retrieval of information through associations formed through Hebbian modification of excitatory connections arising from CA3 (McNaughton and Morris, 1987; Treves and Rolls, 1992, 1994; Hasselmo et al., 1995; Moustafa et al., 2013). The pattern completion of associations in CA3 is enhanced by pattern separation in the dentate gyrus (McNaughton and Morris, 1987; O'Reilly and McClelland, 1994). Associations are formed based on the interactions of hippocampal region CA1 with entorhinal cortex, subiculum and presubiculum (Hasselmo, 2009). Here, we modeled the Hebbian learning through STDP combined with replay. In the sense of the Complementary Learning Systems (CLS) framework (Norman and O'Reilly, 2003) we modeled the memorization of specific events due to replay in the hippocampus regulated by connections from sensory areas to hippocampus and the connections from the hippocampus to motor areas.

Our learning method for the context-dependent task shares with reinforcement learning (RL) (Sutton and Barto, 1998) the credit assignment problem. In RL an agent receives reward after going through a sequence of state-actions, which brought the agent into a goal state. However, some of the actions might not have been necessary, e.g., when navigating there might have been a shorter path to the goal state than the one taken. The problem is now to assign credit only to those state-actions that ultimately led to the goal state. This can be done through temporal difference (TD) learning using eligibility traces and the TD error to update the expected value in a given state. The trace captures the past states and the TD error the state value prediction. Viewed in backwards direction values of all states within the eligible trace are updated through the received reward. Our method solves the credit assignment problem through replay. It keeps a history of states and actions that are part of a trial. This history is long enough to span from any initial state to a goal state, here we have a maximum of two state-action pairs. Then, we replay this state-action sequence in forward order when reward was received and backward order if no reward was received. Through STDP this leads to LTP of connections for rewarding state-action sequences and LTD for state-action sequences without reward.

Previous spiking models have addressed learning of sequences. Brunel (1996) suggested a spiking network using Hebbian synaptic modification to learn a sequence of items in a fixed order. In addition, the network represented the context of items in the sequence, e.g., their successor or predecessor. However, such temporal context is different from the context-specific rule learning, where the rules depend on the context, e.g., in context A the item X is rewarded and in context B the item Y is rewarded (Komorowski et al., 2009).

Hasselmo (2005) suggested a model of prefrontal cortex function to learn goal-directed behavior. In that model, neocortical minicolumns represented sensory input and motor output. Each column had a phase of encoding and retrieval. During encoding STDP was used to learn goal-directed behavior associating states with actions to reach the rewarding goal state. To solve the delayed credit assignment problem, activity spread across bidirectional connections from reward states through the network. The model of Hasselmo (2005) was extended by detailed integrate-and-fire neurons (Koene and Hasselmo, 2005) that simulated properties of unit firing during performance of a cued response task in monkeys (Schultz et al., 2000). The network presented here also uses STDP to learn excitatory connections between the sensory layer and the motor layer, solving the delayed credit assignment problem through a replay mechanism.

Moustafa et al. (2009) uses a rate-based representation and rate-based Hebbian learning rule with one output neuron with different activity levels for different tasks and during different phases of one task to simulate behavioral effects such as latent inhibition, sensory preconditioning or blocking and overshadowing. Learning happens based on an error signal. The adaptation of weights is proportional to the error. Our model uses a spike representation and the STDP learning rule. Each action has its own output motor neuron. Learning occurs during replay in forward or backward temporal order. The adaptation of weights is based on the time difference between pre- and post-synaptic spikes. Our model simulates the learning of a context-based behavioral function matching the learning curve and neural selectivity computed on the spike representation emerging during learning.

The use of both forward and backward replay for learning in the model is inspired by experimental data showing both forward replay (Diba and Buzsáki, 2007; Davidson et al., 2009; Jadhav et al., 2012; Pfeiffer and Foster, 2013) as well as backward replay (Foster and Wilson, 2006; Diba and Buzsáki, 2007; Davidson et al., 2009). Backward replay happens often at the end of a run trial and forward replay happens mostly at the beginning of a trial (Diba and Buzsáki, 2007). Replay has been proposed to mediate formation of associations with reward value (Foster and Wilson, 2006), but there has not been an explicit test of differences in the two types of replay with differences in reward receipt.

### Critical parameters for learning in our model

The model has a few parameters that are critical to the learning of the context-dependent task. The difference between the input currents *I*_*sensory*_, *I*_*hippo*_, and *I*_*motor*_ determine the time differences between pre- and post-synaptic spikes. Since the effective time window of STDP is within the range of 10's of milliseconds these make a strong difference for learning. The necessity of LTD is also expressed through the parameter constraint *A*_−_ < 0. This reduction in weight keeps the weights from saturating. Functional evidence for LTP was found in prefrontal pathway connections to the hippocampus (McNaughton, 1983; Brown and Zador, 1990). Another critical parameter is the inhibition within the model layer 2 and layer 3. This inhibition helps to keep only one neuron active at a time. This prevents “leakage” of activation from prior stimulus activations to the next stimulus activation within an action sequence of one trial. The ratio of the amplitudes of LTP and of LTD is also important for learning. Similar to the data on STDP, our model requires a ratio of *A*_+_/*A*_−_ ≈ 3. The range of weights (*w*_max_ − *w*_min_) is coupled with the amplitudes and time constants for LTP and LTD, these are *A*_+_, τ_+_ and *A*_−_, τ_−_, respectively, and the overall time constant for the synaptic weight change τ_*w*_. When increasing the dynamic range for weights the amplitudes and time constants of LTP and LTD have to be adapted proportionally to guarantee learning of the context-dependent task. Finally, for reasons of numerical stability the step width Δ*t* has to be sufficiently small. This step width interacts with all input currents and voltage values. Larger currents and voltages require smaller Δ*t*'s.

### Tasks related to context learning and abstraction

Similar context-dependent tasks analyzed brain areas involved in the acquisition and use of conceptual knowledge. Kumaran et al. (2009) used a weather forecast task where the participant had to predict sun or rain depending on the displayed object or the location in which an object appeared. In some trials location determined the correct response and in other trials the object type determined the correct response. Probe trials designated as “determined” used a determining object type or location, while undetermined probe trials used objects in locations so that object and location coded different correct responses. Parahippocampal cortex, amygdala, posterior cingulate cortex, ventral striatum, and ventromedial prefrontal cortex (vMPFC) showed activity that correlated with success likelihood during learning. The activation in the hippocampus and vMPFC was significantly greater for determined probe trails than for undetermined probe trials. This data suggests that vMPFC is involved in the acquisition of conceptual knowledge. In the context-dependent task used here all stimuli were determined, however, the rat could generalize the rule inversion with a context switch (Komorowski et al., 2009).

Badre et al. (2010) use in their task ask for an abstract label 1, 2, or 3 as response, which depends on the shape, orientation, or a colored frame surrounding a presented figure. Two sets of three object shapes, which can appear in three orientations and are surrounded by one of two colored frames, are used to define the stimuli (2 × 3 × 3 × 2 = 36 combinations). Their study focused on the learning of hierarchical rules. In one setting the learned rule is flat; an association between shape, orientation, color, and response has to be learned. In another setting the rule is hierarchical where the color indicates if the shape or orientation information determines the response. During learning, rostro-caudal frontal brain regions were activated. Teasing apart the learning of flat and hierarchical rules shows an early activation in pre-premotor cortex. The learned representation for the context-dependent task did not involve a hierarchy. However, the task itself could be expressed containing a hierarchy in the following way. The first level in the hierarchy simply expresses the mapping between items and reward. The second level in the hierarchy represents the different mappings given for different contexts. In the context-dependent task, the mapping is inverted with each context switch.

Wallis et al. (2001) used a delayed match to sample task. A sample picture appears followed by a match picture after a delay. During implementation of one rule the monkey has to pull a lever if the pictures match. During implementation of another rule the monkey has to pull a lever if the two presented pictures do not match. The content of the pictures is irrelevant to the task as only the matching or non-matching matters. Single neurons in the PFC encode these two abstract rules. In the context-dependent task the same items were always presented, defined either through, visual, tactile, or olfactory stimulation within one experiment. The only abstraction is the rule switch between contexts. Our spiking model did not focus on abstract rule learning, which we assume to be solved by e.g., a hierarchical network. In this hierarchy one layer would define items as the same input if they are the same or distinct input if they differ. Another layer would take this input signal about items to learn the rules independent of the item identity. Testing such a hierarchical model with the matching/non-matching task is future work.

### Conflict of interest statement

The authors declare that the research was conducted in the absence of any commercial or financial relationships that could be construed as a potential conflict of interest.
